# Complete remission in a rare pelvic angiosarcoma with liposomal doxorubicin: A comprehensive case report and review of literature

**DOI:** 10.1177/20363613251324973

**Published:** 2025-02-27

**Authors:** Truls Gråberg, Andri Papakonstantinou, Felix Haglund de Flon, Ivan Shabo, Ann Morgell, Christina Linder-Straglitto, Inga-Lena Nilsson, Fredrik Karlsson, Robert Bränström

**Affiliations:** 1 Department of Clinical Intervention and Technology, 27106 Karolinska Institutet, Stockholm, Sweden; 2 Department of Molecular Medicine and Surgery, 27106 Karolinska Institutet, Stockholm, Sweden; 3 Department of Breast, Endocrine Tumors and Sarcoma, Karolinska University Hospital, Stockholm, Sweden; 4 Department of Oncology-Pathology, 27106 Karolinska Institutet, Stockholm, Sweden; 5 Department of Pathology and Cancer Diagnostics, Karolinska University Hospital, Stockholm, Sweden

**Keywords:** Angiosarcoma, doxorubicin, surgery, long-term follow-up

## Abstract

Angiosarcoma, an aggressive sarcoma subtype originating from lymphatic or vascular endothelial cells, is rare, constituting less than 2% of all soft tissue sarcomas. Predominantly affecting adult and elderly patients, it manifests diversely across various anatomical locations, with cutaneous lesions being the most common, particularly in the head and neck region. Noteworthy for its infiltrative nature, angiosarcoma demonstrates high rates of local recurrence and metastasis, leading to poor overall survival. The diagnosis may be difficult due to nonspecific clinical symptoms, and histological examination is essential in this disease. Treatment typically requires radical surgery, with addition of either chemo- or radiotherapy, or occasionally both, but there is a lack of formal evidence for the order of the modalities employed. Emerging therapies, such as targeted medicines and immunotherapy, show promising results in improving outcomes. This report presents a comprehensive analysis of a rare case of a young male with pelvic angiosarcoma. The patient underwent multiple operations, chemotherapy, and radiation, which highlights the complexities in management and the need for a multidisciplinary approach. Despite challenges, the patient achieved complete remission and is disease-free over 16 years after pelvic exenteration, demonstrating the potential for successful long-term outcomes. The case underscores the importance of personalized, multimodal treatment plans and close collaboration between surgeons and oncologists. Continued research into tailored therapies offers hope for improved prognosis and quality of life for individuals facing this uncommon sarcoma.

## Introduction

Angiosarcomas are uncommon and highly aggressive tumors that originate from lymphatic or vascular endothelial cells. This type of sarcoma constitutes less than 2% of all soft tissue sarcomas in humans,^1^ and it predominantly affects adults and the elderly.^1,2^ Angiosarcomas are a diverse subgroup of sarcomas that can manifest in various locations throughout the body.^3^ Cutaneous lesions, accounting for approximately 60% of cases, are most prevalent, especially in the head and neck region.^1^ However, angiosarcomas can also occur in soft tissues, visceral organs, bone, and the retroperitoneum.^1,3^

Noteworthy for its infiltrative nature, angiosarcomas exhibits a high rate of local recurrence and metastasis.^1,4,5^ At presentation, rates of advanced or metastatic disease range from 16% to 44%, and median overall survival is reported to be 16 months.^4^ The pathogenesis of primary angiosarcomas remains not entirely understood, but definite risk factors include chronic lymphedema, exposure to environmental carcinogens (such as vinyl chloride, thorium dioxide, and arsenic), and certain genetic syndromes.^1,2,6^ Angiosarcomas are usually subdivided into primary, and radiation (or rather radiotherapy) associated angiosarcomas. The incidence of the latter is rising, and it affects approximately every one-thousandth patient having received adjuvant radiotherapy after breast cancer surgery.^7–9^ Epidemiological studies show a relatively equal distribution between genders, although cutaneous angiosarcoma tends to exhibit a notable preference for older male individuals, with a reported median age in the sixth decade.^10^

Diagnosing angiosarcoma may be difficult due to its nonspecific symptoms, making it challenging to distinguish it from other malignant neoplasms, e.g. anaplastic melanoma and epithelial carcinomas.^2,11,12^ While ultrasound, computed tomography (CT), and magnetic resonance imaging (MRI) may aid in making the diagnosis, they have limitations. Therefore, histological examination is crucial for diagnosing angiosarcomas, frequently requiring immunohistochemical confirmation.^5,13^ Histologically, angiosarcoma has a wide histomorphological spectrum ranging from well-differentiated infiltrating vessels with inconspicuous atypia and multilayered endothelia, to poorly differentiated solid sheets of atypical spindled, polygonal or epithelioid cells lacking vasoformation. Tumor cells show expression of vascular antigens on immunohistochemistry, including CD31, CD34, FLI1, VEGF, D2-40 and ERG, and the epithelioid subtype express cytokeratins and EMA.^1,2,11,12^

The diagnostic difficulties and the rarity of angiosarcomas contribute to challenges in determining the optimal treatment and prognostic factors. The optimal approach includes radical surgery.^2,5,11^ Neoadjuvant or adjuvant chemotherapy or radiotherapy can be considered in selected cases, given the high metastatic potential, although evidence is scarce.^14–16^ In inoperable and metastatic cases systemic therapy with anthracyclines, taxanes or gemcitabine regimens are recommended. Moreover, targeted therapy with pazopanib and immune checkpoint inhibitors have shown promising results, especially in cutaneous angiosarcomas, and can be offered to selected patients.^17–19^ The multifaceted nature of angiosarcoma necessitates a comprehensive understanding of its clinical characteristics and treatment modalities for improved patient outcomes.

In this study, we aim to provide a comprehensive analysis of a rare case involving a young male who presented with a primary angiosarcoma in the lesser pelvis. Additionally, we thoroughly review the current literature on angiosarcomas to enhance our understanding of the clinical characteristics and management strategies associated with this uncommon malignancy.

## Case report

**Figure 1.**
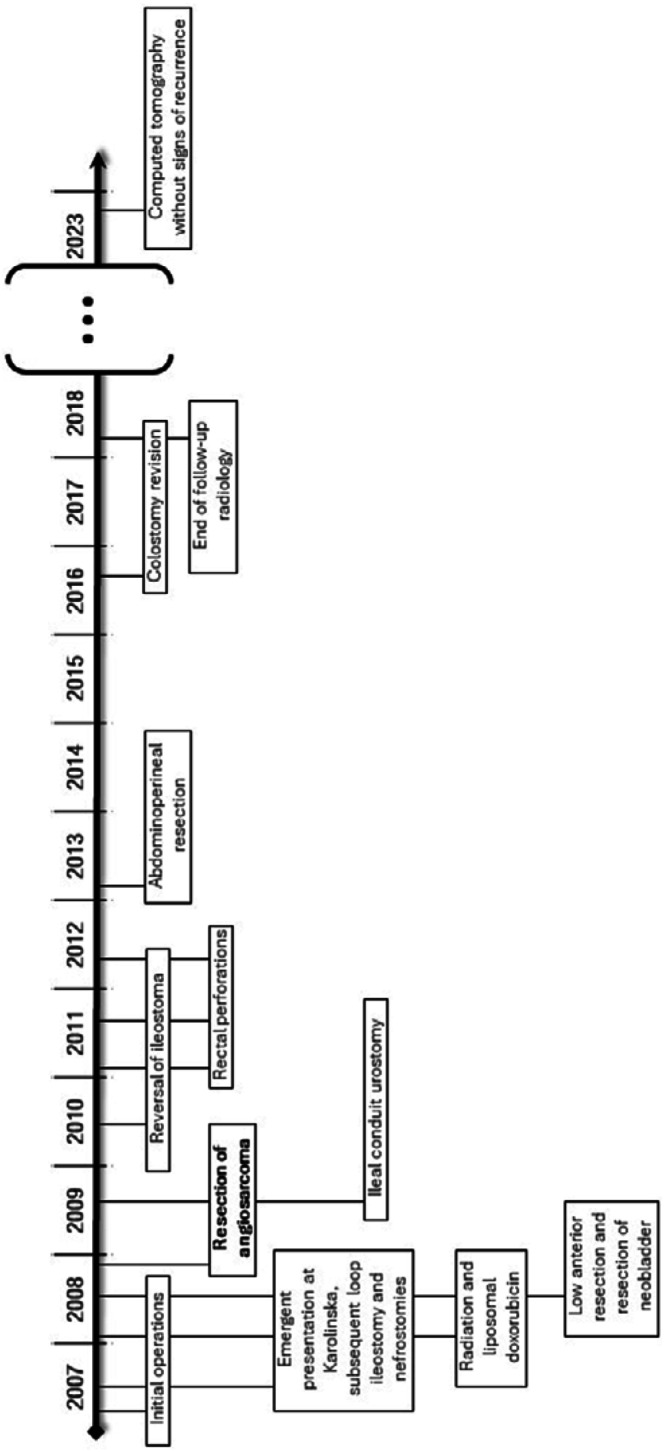
Timeline illustrating the significant events during the patient’s medical journey, from diagnosis in 2007 to long-term follow-up.

A previously healthy man was diagnosed with, and subsequently operated on, for epithelioid angiosarcoma of the seminal vesicle at the age of 28 years. The operation, which was carried out at another university hospital, was an initial laparoscopic resection of the left seminal vesicle. Upon receiving the histological diagnosis of epithelioid angiosarcoma, resected with positive margins, the patient underwent another operation at the other hospital. This included a cystectomy, retroperitoneal lymphadenectomy, resection of a short segment of the small intestine and construction of an orthotopic neobladder. A case timeline is presented in Figure 1.

The young man presented at Karolinska University Hospital 2 months and two and a half weeks after the second operation with a fever and fistula from the neobladder was diagnosed. During an exploratory laparotomy, signs of residual or recurring angiosarcoma almost filling the pelvis were found and confirmed in multiple biopsies.

**Figure 2.**
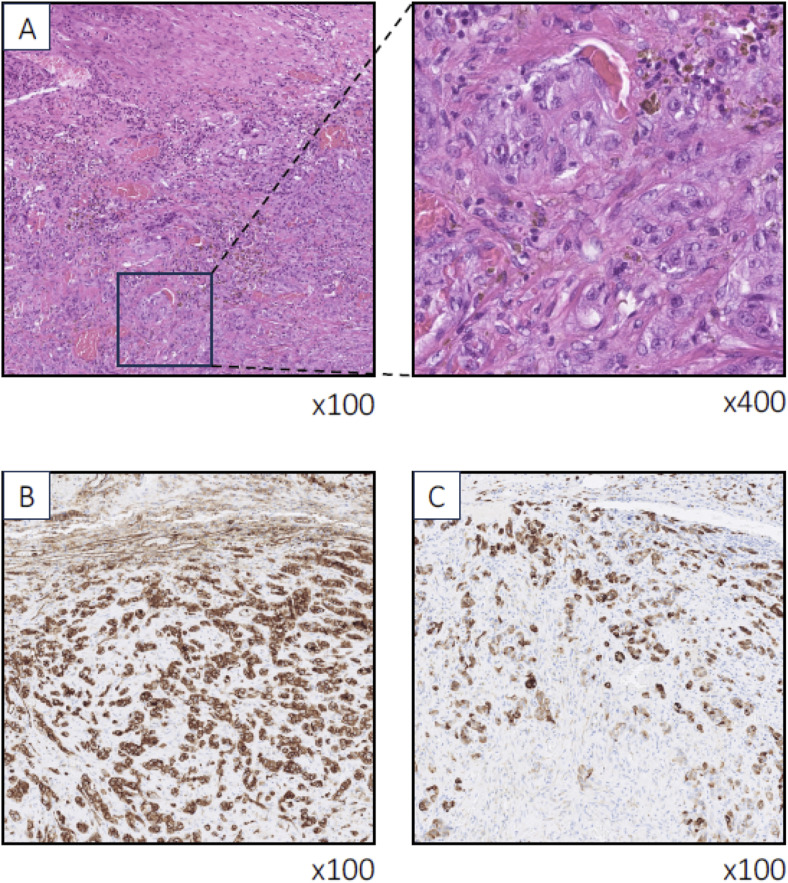
Photomicrographs displaying the initial peritoneal metastasis. In the top panel (A), infiltration of markedly atypical epithelioid tumor cells is evident, forming immature vascular structures, some of which contain erythrocytes. Immunohistochemical analysis confirms the presence of the vascular marker CD31 (B) and pan-cytokeratin AE1AE3 (C) in the tumor cells. Immunohistochemistry for FOS, FOSB, TFE3, and CAMTA1 was negative in the tumor cells (not shown). Magnification levels are indicated as ×100 and ×400.

Immunohistochemical staining confirmed the presence of the vascular marker CD31 and pan-cytokeratin marker AE1AE3 in the tumor cells (Figure 2). The tumor cells did not exhibit expression of FOS, FOSB, TFE3, or CAMTA1, thereby excluding epithelioid hemangioma, pseudomyogenic hemangioendothelioma and epithelioid hemangioendothelioma. An enterostomy was made, and bilateral percutaneous nephrostomies were placed the following day.

After assessment by an experienced oncologist, a chemotherapeutic regimen with liposomal doxorubicin at a dose of 30 mg/m^2^, every 4 weeks, was initiated. The patient had previously received radiation therapy; 5 sessions of 4 Gy, a total dose of 20 Gy. Initially, it was deliberated combining liposomal doxorubicin with Bevacizumab but considering previous multiple operations and the radiotherapy recently given, the risk of intestinal perforation was deemed to grave. Similarly, the first dose of liposomal doxorubicin was reduced to 75%. After 5 doses of this course of chemotherapy, and approximately 1 year after the patient presented at our hospital, another operation was necessary to bring recurring bouts of infection, arising from a colo-neovesicular fistula, under control. At this operation the neobladder was partially resected, and the fistula between the sigmoid colon and the neobladder was closed. At that point, there were no macroscopic signs of disseminated tumor, i.e. no peritoneal carcinomatosis or liver metastases. There were no signs of cardiotoxicity as assessed by repeated echocardiography, during or after the course of liposomal doxorubicin. Throughout the chemotherapeutic treatment, as well as before and after this and following operations, the patient’s nutrition was managed by varying combinations of parenteral, enteral and peroral supplementation, managed in close cooperation with clinical nutritionists. After recovery from this operation the chemotherapeutic regimen was resumed.

**Figure 3.**
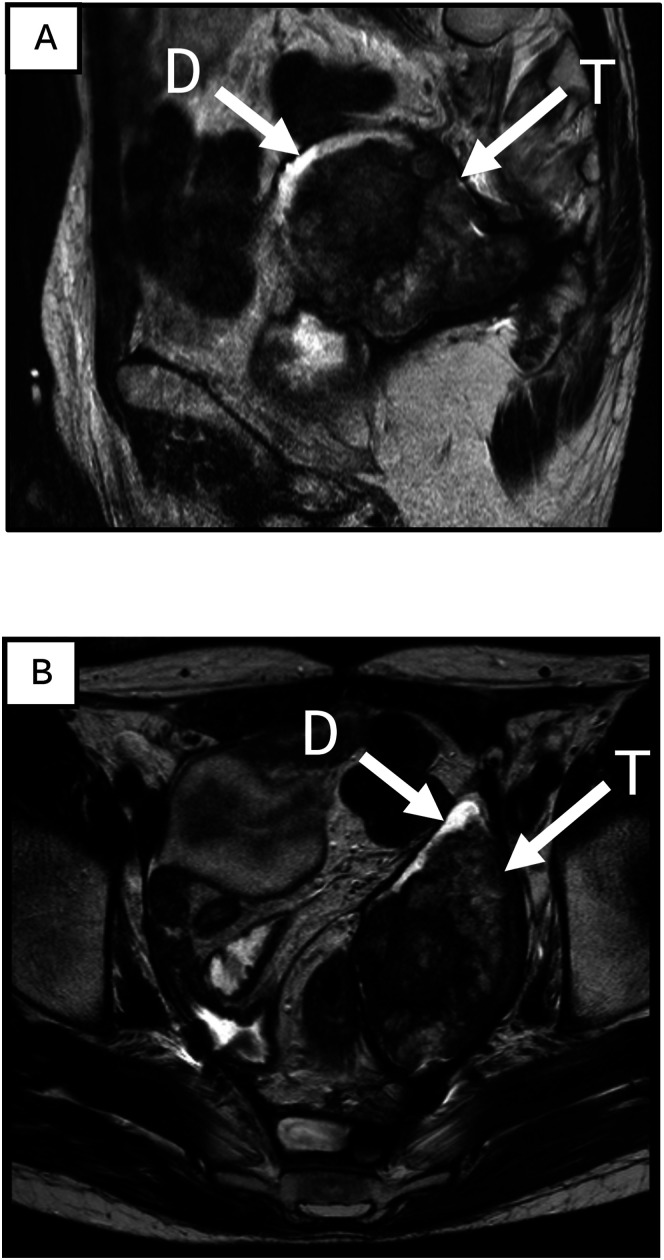
Magnetic resonance tomography images displaying the pelvic tumor from sagittal (A) and transverse (B) views. The tumor is indicated by a white arrow labeled ‘T’. Additionally, denser tissue observed in the anterocranial region corresponds to residual metabolic activity detected in a Positron Emission Tomography (PET) scan, shown as ‘D’.

Upon completion of the pre-operative course of chemotherapy, consisting of a total of 11 doses of liposomal doxorubicin, follow-up CT and MRI scans demonstrated significant regression of the pelvic mass, as shown in Figure 3. A Fluorodeoxyglucose (^18^F) positron emission tomography (FDG-PET/CT) showed a slight remaining metabolic activity corresponding to a crescent of more dense tissue in the periphery of the mass. At a multidisciplinary treatment conference, 2 years after the first contact with the patient at this hospital, it was decided to offer the patient another operation to remove the tumor mass. The decision was based on a multidisciplinary assessment of benefits and risk. Among other considerations it was found that the response to the chemotherapy of the pelvic mass had reached a standstill. The patient consented and was operated on with a low anterior resection, including a wide excision of soft tissue in the pelvis. The previously fashioned enterostomy was kept, and a colorectal anastomosis was made with a circular stapler. The histological examination of the specimen from this operation revealed no remaining viable tumor cells. Following the patient’s recovery after this operation, a consolidating course of three additional cycles of liposomal doxorubicin were given over a period of three and half months. This was given, after multidisciplinary evaluation, based on no remaining viable sarcoma cells in the specimen from the operation. In total, 14 doses of liposomal doxorubicin were given. 10 months later, a Bricker conduit was fashioned, replacing the bilateral nephrostomies. 10 months after that, the enterostomy was reversed.

Three re-operations followed, mandated by radiation-induced perforations of the rectum. Once the patient recovered, an abdominoperineal resection of the rectum and the creation of a permanent colostomy were performed to eliminate the risk of future perforations. Nine years after his initial presentation at this hospital, his stoma was revised to manage recurring episodes of protrusion.

In end-effect, the patient received a pelvic exenteration, barring parts of the prostate. He has undergone follow-up CT and MRI-scans, initially quarterly and later biannually, up until approximately 10 years from diagnosis, without any signs of recurrence. A CT-scan, undertaken in another hospital, on the suspicion of small bowel obstruction, demonstrated no signs of recurrence, 15 years and 9 months from the initial surgery. This small bowel obstruction resolved spontaneously, and this is the only hospital admission the patient has had over the last 5 years. As of recently, close to 17 years after the diagnosis, the patient is without recurrence and employed full-time as a metal worker.

## Discussion

Angiosarcomas, a rare and aggressive malignancy arising from lymphatic or vascular endothelial cells, presents a unique set of challenges in the oncology landscape.^1–3^ Its diverse manifestations add complexity to its diagnosis and management. Despite extensive research efforts, the precise etiology of angiosarcoma remains elusive. While certain risk factors have been implicated, cases like the one presented here highlight instances where no identifiable risk factors are present, emphasizing the need for further exploration into the underlying mechanisms driving tumorigenesis.

Diagnosing angiosarcoma poses a significant challenge due to its rarity, often leading to delays in diagnosis and subsequent initiation of correct treatment. Treatment decisions are often complicated by the advanced stage at which many cases are diagnosed, due to the infiltrative nature of angiosarcomas. Therefore, there exists an urgent need for improved diagnostic modalities.

Current treatment strategies for angiosarcoma ideally encompass a multidisciplinary approach, in which it should be considered including radical surgery, radiotherapy, and chemotherapy. Also, emerging targeted therapies and immunotherapy hold promise for improving outcomes and enhancing patient survival rates. However, owing to the rarity of primary angiosarcomas, treatment studies and trials are not easily carried out. The case presented here, involves a young male with pelvic angiosarcoma, and serves as a poignant reminder of the importance of a comprehensive and personalized treatment approach, necessitating close collaboration between surgical, medical, and radiation oncologists.

Challenges characterize many patients’ journeys through the landscape of angiosarcoma treatment. From the initial laparoscopic resection to subsequent surgeries, chemotherapy sessions, and radiation therapy, the course of the case presented was fraught with adversities. Nevertheless, the patient’s remarkable response to liposomal doxorubicin and the subsequent surgical interventions underscore the resilience and determination required in treating this rare tumor entity. The patient’s enduring disease-free interval of over 16 years is a testament to the need for multidisciplinary management at a specialist center, highlighting the potential for long-term survival and quality of life improvements in patients diagnosed with angiosarcoma. Data on efficacy of liposomal doxorubicin in angiosarcomas, not least in a preoperative setting, are scarce. Retrospective data from Italiano et al.,^20^ show that 20 out of 34 patients with metastatic angiosarcoma treated with doxorubicin had clinical benefit, of which two (6%) had radiological complete response. Our case, further highlights the benefit of anthracycline-based therapy, confirming its effect at the pathological level.

## Conclusion

Angiosarcoma remains a formidable oncological challenge, necessitating a concerted effort to elucidate its underlying mechanisms and develop treatment strategies. The presented case underscores the importance of accurate diagnosis and subsequent tailored treatment plans with a panoptic consideration of therapeutic approaches. Moving forward, advancements in diagnostic techniques, treatment modalities, and other interventions promise better quality of life and improved survival rates for patients diagnosed with angiosarcoma.
